# Isolation, Solitude and Social Distancing for People Who Use Drugs: An Ethnographic Perspective

**DOI:** 10.3389/fpsyt.2020.623032

**Published:** 2021-01-13

**Authors:** Laura Roe, Jesse Proudfoot, Joseph Tay Wee Teck, Richard D. G. Irvine, Stan Frankland, Alexander Mario Baldacchino

**Affiliations:** ^1^Department of Social Anthropology, University of St Andrews, St Andrews, United Kingdom; ^2^Department of Sociology, Durham University, Durham, United Kingdom; ^3^School of Medicine, University of St Andrews, St Andrews, United Kingdom

**Keywords:** substance use, COVID-19, isolation, solitude, social distancing, substance use disorder, harm reduction

## Abstract

COVID-19 has resulted in deepened states of crisis and vulnerability for people who use drugs throughout Europe and across the world, with social distancing measures having far-reaching implications for everyday life. Prolonged periods of isolation and solitude are acknowledged within much addiction literature as negatively impacting the experiences of those in recovery, while also causing harm to active users – many of whom depend on social contact for the purchasing and taking of substances, as well as myriad forms of support. Solitude, however, is proposed by the authors as inherent within some aspects of substance use, far from particular to the current pandemic. Certain forms of substance use engender solitary experience, even where use is predicated upon the presence of others. Adopting a cross-disciplinary perspective, this paper takes as its focus the urgent changes wrought by the pandemic upon everyday life for people who use drugs, drawing on recent ethnographic fieldwork with substance users in Scotland. Beyond the current crises, the paper proposes solitude, and by extension isolation, as an analytical framework for better apprehending lived experiences of substance use.

## Introduction

Considerations of isolation and solitude in relation to Substance Use Disorders (SUD) are often accompanied by portrayals of life as lacking in social connection, where the possibilities for meaningful relationships are subsumed by the compulsive drive toward substances. In popular depictions, bonds of family and friendship are turned away from and the person gradually finds themselves alone, together only with the substance. Characterisations such as these have extensive roots in historical understandings of addiction, with substance use and social relationships having been positioned as mutually exclusive since at least the late 1700s (1). Contemporary addiction scholarship has done much to dispel such notions, with anthropological and ethnographic works exploring social bonds as profound components of substance use, or else as being minimally affected by it (2–4). Everyday survival for some people who use drugs frequently depends upon the maintenance of (often fragile) social networks – with certain forms of use entailing specific configurations of relatedness, intimacy, and care.

The Covid-19 pandemic has made such survival strategies even more precarious. Social distancing measures have, for instance, created ripple effects on drug supply chains; the ability of individuals to procure desired drugs and injecting equipment, and the operation/accessibility of harm reduction and healthcare services, amongst many other unintended consequences (5–7). Fluctuating availability of substances is likely to affect individual tolerance and may lead to increased risk of overdose (8). Some intervention, harm reduction, and recovery services have faced significant challenges in transitioning from in-person contact to online and phone support (9). Some have increased the amount of allowable unsupervised take-home opioid replacement therapy to facilitate social distancing measures, whilst others have flooded the market with Take Home Naloxone (THN) (7, 10, 11). Intensive experiences of isolation, known to be detrimental to well-being and recovery, have been exacerbated by the pandemic, with disastrous effects on the mental and physical health of many people who use drugs (12). Through its threats to social bonds and relatedness, therefore, the pandemic has deepened the difficulties encountered by many on a day-to-day basis.

And yet, alongside its inherently social aspects, substance use is also intimately connected to solitude, whether that be in everyday moments such as the high, or distilled across longer spans of time, so that life itself comes to feel solitary. Solitude therefore occupies an essential place within substance user sociality, coming to be connected with experiences of loneliness, boredom, emptiness, and senses of time as endless or repetitive. At the same time, it is important to recognize that the experience of solitude can be sought-after, and that this itself can provide a motivation of substance use. Recent theorisation of solitude has challenged its inevitable characterization as pathological (13), highlighting the importance of time spent alone for well-being (14, 15). Moreover, collective desire for solitude can itself be a source of shared experience (16). It is therefore necessary to distinguish between solitude as deliberate withdrawal, and isolation as an involuntary loss (or non-existence) of social ties, implying an absence of or alienation from social relationships. The state of being alone can be intentional or unintentional; destructive or restorative; situational or existential [c.f. (17)]. The challenge of theorizing solitude for people who use drugs is how to explore it as commonplace while avoiding tropes of substance use as inherently and inevitably isolating. This paper seeks to address the question of how isolation and solitude can form an analytical framework to better explore the experiential, temporal, and material impacts of the pandemic – and social distancing – on people who use drugs.

## Solitude as Social Distancing

Social bonds between people who use drugs are often characterized as being governed by need – predominantly financial or logistical – with SUDs viewed as compromising the trust, intimacy, co-operation, and care that characterizes close relationships. Ethnographic studies of people who use drugs, however, offer countless examples of relationships that easily fit into “normative” configurations of sociality and friendship and extend far beyond pragmatism, though like all relationships they might at times be mediated by self-interest, self-preservation, and necessity [c.f. 2, 4]. Under “conditions of scarcity,” people who are dependent on drugs must often carefully negotiate self-preservation against the possibility of becoming socially isolated (2). For such users, isolation from one's social network often entails heightened risk of withdrawal, as procuring substances becomes much more difficult (18); the risk of overdose is increased if one uses alone (19); and there are myriad harms associated with economic and social precarity (20–22). Social bonds therefore offer protection against the dangers of isolation, although relationships based around substance use cannot be reduced to pragmatic aspects, nor be easily generalized. Aaron Goodfellow (4), for instance, notes that although heroin use was almost always a central aspect of relationships between heroin users, relationships were not inexorably defined by it. While social bonds could be fragile and fractious, relationships between heroin users comprised unanticipated, novel, and at times “normative,” forms of relatedness that offered profound senses of meaning, purpose, and fulfillment.

Isolation can, as such, lead to increased or riskier substance use, partly because the social relations and interactions that offer purpose and meaning are unattainable. In addition to the harmful effects of isolation mentioned above, too much time on one's own can foment powerful senses of boredom, anxiety, and loneliness, as well as precipitate the return of painful memories, against which substances provide a means of relief. For those in recovery, too, the draw toward substances is often coupled with boredom, loneliness, and feelings of hopelessness, with everyday life ceasing to feel meaningful. Diverse studies on boredom (23–35); waiting (25, 26, 28, 31, 36); and notions of being “stuck” (34), draw attention to the distinctly temporal dynamics that characterize such experiences, many noting in particular that future hopes and aspirations appear inaccessible. Clouded senses of the future are further exacerbated by economic precarity (24, 28–30, 34) and experiences of subjugation (31, 33) that provoke a sense of the present as endlessly, and inescapably, repeating. Temporal repetition can easily become oppressive and anxiety-inducing, leading to “thinking too much” and being overwhelmed by difficult or distressing memories (26, 33, 34).

## Case Study

This was often the case for many of the authors' research participants, across a variety of research settings. ^1^ During Roe's ethnographic research, which largely took place in 2017 in an East coast county in Scotland, themes of isolation, boredom, anxiety, and senses of time as endlessly repeating arose in dozens of interviews and conversations with people who used drugs (37). Accompanying a small number of individuals in their everyday lives over a period of several months further illuminated the connection between isolation, the affective-temporal states isolation occasioned, and participants' continuing substance use.

One such participant was Tamsin, a woman in her early thirties who had been using heroin since the beginning of her twenties, and a variety of other substances since she was a young teenager.^2^ Tamsin described having made countless attempts to become abstinent over approximately 5 years, which accompanied a fluctuating engagement with various recovery and counseling services. Although she mentioned having had a methadone prescription in the past, she was not involved with NHS Addiction Services during the time of the research.

For Tamsin, the stresses of everyday life could be moderated through specific forms of substance use, usually in which heroin was combined with various other psychoactive substances, such as benzodiazepines and alcohol. Although Tamsin also frequently used heroin on its own, she described its effects as minimal and unobtrusive. By comparison, the intoxication she sought through poly-drug use had potent effects on her consciousness, emotional state, and perception of time. Tamsin often described how feeling bored, isolated, and alone often led to escalated use of heroin and other drugs.

The below conversation with Tamsin took place during Roe's initial research, during a casual visit to a park near her home. It was one of many in which Tamsin described isolation and boredom as both “claustrophobic” and connected to specific forms of substance use (37).

Laura: Do you mind being on your own?

Tamsin: Not really, like I can be by myself, I'm not one of those people that cannae stand being alone.^3^ Ken I don't mind my own company, most of the time, but it just gets kind of boring sometimes.^4^ Especially if you're not in a good place, like, mentally. [Laughing] The walls start closing in.

Laura: How do you mean?

Tamsin: Well, like, if you're feeling shit about things anyway, nothing's happening, folk are being cunts. I start feeling claustrophobic, especially if I'm trying to keep aff it.^5^ Start thinking about shit.

Laura: Like what kind of stuff?

Tamsin: Dunno, just stuff. Bad things, traumatic shit. I think it's why I use.

Laura: Do you think you use when you get bored?

Tamsin: I use because I have to, mostly, but aye, I guess boredom is a big thing. It's good for killing time. […] Everything's the same, day in, day out; same old shit.

Laura: That makes sense. Does it make time pass quicker, do you think, or do you just stop noticing it? The time, I mean.

Tamsin: [hesitating] I'm not sure. It depends, maybe. Downers like Vallies just slow everything right down, in a nice way though.^6^ Relax you. […] Things just fall away, ken, nothing matters.

Tamsin illustrates above that senses of being alone with nothing to do – along with resulting feelings of boredom and entrapment – can be countered with specific substances. Tamsin gestures toward the return of traumatic or painful memories in such circumstances, which she here implies are distanced with substance use – temporarily forgotten or remembered less sorrowfully. Similarly, there were other occasions in which Tamsin described her use of substances as removing her from the present, which was often experienced as stressful, dull, and repetitive. In this context, substances can be used to forge new affective-temporal scripts, in which the slowness and emptiness of time – rather than being laborious and intolerable – is dwelled in, and other concerns or crises are rendered second to the high. At other points, Tamsin noted that getting high provided breathing space from daily life, a sentiment echoed by several other participants in the research. This desirable form of solitude differed from isolation, answering a desire that Tamsin once framed by saying “I just want to get away from everyone and everything.” Substances could therefore counter the negative aspects of isolation by enabling a more peaceable sense of aloneness and solitude and, perhaps paradoxically, by enabling one to “isolate” oneself from trauma, pain, and boredom. Tamsin gives a sense of retreating into herself and into alternative temporalities, evoking a form of social distancing that would seem to give new meaning to the term. The space created from others was not merely physical, as we have come to understand by “social distancing,” but rather a means of inhabiting, albeit temporarily, a world of one's own.

Natascha Dow Schüll (38) makes a similar observation in her research with compulsive “slot,” or “fruit” machine gamblers. While popular representations of gambling often focus on gamblers' desperate attempts to turn around a losing streak by “winning big,” or the thrill of risking it all on a single turn of the wheel, Schüll notes that what machine gamblers value most of all is the solitude of what they call “the zone”: a state of detachment from everyday life that they experience when immersed in the machine. In this space of predictable, pleasurable repetition, the messiness of human relationships and caring responsibilities melts away, replaced by solitary communion with the machine. Such desires for solitude, understood as a means of managing the problem of being with others, and the pursuit of activities that transform solitary experience into something pleasurable which steps out of time, have striking parallels with the solitude many of our respondents describe pursuing in drugs.

Without undermining the sociality of using substances, and the myriad forms of relatedness made possible through substance use, solitude come to the fore as a meaningful and sought-after experience (35). Even where substances are taken in the company of others, moments of solitude can be achieved through the high. It should also be acknowledged that isolation and solitude can be sought outside of taking substances and becoming intoxicated, and instead permeate everyday life. Substance users also spoke of isolating themselves from family, friends, and other social contact even where it was known to be destructive or detrimental to their health and well-being. Tamsin, for instance, notes the ability of substances to make “things fall away” but at other times gestured toward the satisfaction to be found in simply “letting things fall apart” (37).

Isolation and solitude overall have multiple dimensions, both negative and positive, that are avoided and pursued to varying extents by people who use drugs. Social relationships, substance use, and solitude can each serve as intertwining buffers to the harmful aspects of isolation. In the context of Covid-19, in which isolation presents genuine threats for people who use drugs, even dangerous patterns of poly-substance use can be understood as specific means of countering experiences of isolation, loneliness, frustration, boredom, and despair.

## Isolation and Solitude in Covid-19

Social isolation for people who use drugs takes on new significance in light of the pandemic, particularly in the face of nationwide lockdowns and social distancing measures. In certain cases, actions taken to prevent the spread of the virus have been beneficial to substance users – such as services delivering essentials of food and medication to users who are shielding – while other measures have worsened the difficulties faced by users on a day-to-day basis (39). Homeless substance users offered temporary accommodation for the duration of the crisis, for instance, have found themselves facing intensified isolation, due to being removed from social networks and known spaces (40). Smith (41) similarly notes that homeless people in London were disadvantaged by their relocation to different parts of the city, away from the familiar terrain and social relationships that offered access to things like food and companionship. Some even favored rough sleeping over private lodgings. The authors noted similar trends in working with homeless substance using populations in Scotland (although there were equally those that enjoyed the sociability of shared accommodation, as opposed to the isolation of sleeping rough) (42). These challenges are by no means new, but are nonetheless greatly exacerbated in the current context, wherein already precarious social relationships and circumstances are further fractured and destabilized.

Isolation during Covid-19 has also been noted in numerous emerging studies as leading to both increased and riskier substance use, and frustrating attempts to recover (43–47). In a survey conducted by the Scottish drug treatment and education charity Crew, isolation, boredom, and stress were cited as reasons for increased substance use in Scotland, with 58% of 300 participants reporting an increase in their use (44). A survey undertaken by the New Zealand Drug Foundation, similarly, found increases in use – boredom and anxiety being reasons most commonly given – although lockdown enabled some to reduce their use (45). A recent study by the Well-being Trust cites isolation, stress and financial hardship as significantly increasing the likelihood of higher drug-related deaths [c.f 47]. Issues of isolation have been further compounded by major disruptions to drug supply chains across the world, with decreased availability and increased prices prompting the use of alternative substances – in turn heightening the risk of changes in individual tolerance and overdose (7, 46).

The authors have observed similar shifts in patterns of substance use in Scotland, including, for example, a significant rise in the use of crack cocaine, benzodiazepines, and alcohol. In discussions with active and recovering substance users, deepened senses of isolation, loneliness, anxiety, and boredom – combined with fluctuating availability of substances – acted as catalysts for more frequent and more chaotic using.

## Case Study

In a series of phone conversations in May, June, and July of this year, several of Roe's research participants discussed the everyday difficulties occasioned by lockdown restrictions and social distancing measures. During one such phone call, Tamsin detailed her own experiences of lockdown, describing overlapping senses of isolation, boredom, and anxiety as harming her efforts to remain abstinent.^7^ In response to being asked about her experiences during the pandemic, she had answered:

I was totally climbing the walls, like. It drives me a bit mental, being cooped up. I need to be doing things or else I just get bored… Aye, I was still going out to score, but I was so anxious about it, ken, I wouldn't have done it if I couldn't of. I was at my mum's for a bit, but it was hard going. I stayed with my pal, but we fell out when she fucking robbed me. […] I've not really been seeing anyone, ken, trying to stick to the rules and everything. It's hard going, like. I was doing okay before, but this knocked me back, like my anxiety is a lot worse. […] I slipped near the beginning, and it's fucked me. Never ends, eh?

The conversation meandered through a number of other topics, eventually progressing to the impact of the pandemic on local drug supply. On this matter, Tamsin described a lack of available heroin and the need to resort to alternative substances:

I couldn't get heroin a few times, but I got crack, eh, just to stop me fae rattling.^8^ The heroin's been shit quality. There was fake Vallies going about too. I was injecting crack, so my veins are probably fucked. More fucked. […] I never liked crack, I never really got high off it. I was on my own, which wasn't the best idea, probably. My drug debts are a lot worse too, it's stressful.

The conversation then turned to the effects of substances and, when prompted, Tamsin reasoned that substances permitted a sense of uncertainty toward the negative aspects of isolation and the overall stresses of the pandemic:

My using's been pure chaotic lately, but I've been that stressed. […] It just helps you get away from it all, ken. I don't know, I don't have to think. It kind of just gets you out of what's happening.

From conversations with Tamsin and a number of other substance users, it became apparent that the isolation experienced during and after lockdown both highlighted and exacerbated pre-existing difficulties and crises: “You just feel, like, what's the point? […] Things just always seem to get worse” as Tamsin put it. Substances continued to offer Tamsin a means of “self-isolating” or “shielding” from trauma, anxiety, and boredom, which were worsened in this case by her literal self-isolation.

Tamsin's experiences are of course specific to her own situation, although it is notable that much of what arose in the above phone call was echoed by other substance users. Four other individuals spoken to by Roe during this time emphasized the worsened precarity of their living situations; the fluctuating difficulty of obtaining regular substances; the need to resort to alternative substances to avoid withdrawal; and the use of substances to counter the adverse effects of prolonged isolation, boredom, and anxiety on their mental health and well-being. These issues are far from isolated to substance users in Scotland, but have been evidenced in several recent international studies on the impacts of the pandemic upon substance using populations. Banducci and Weiss, for example, in working with individuals with posttraumtic stress disorder (PTSD) and SUDs in the US, observed that substances were used to counter isolation, stress and the “monotony” of daily life under stringent social distancing measures (48). The turmoil wrought by the pandemic, they go on to argue, complicates recovery in part by rendering the future uncertain. Researchers based in a residential treatment center in Los Angeles similarly reported that treatment retention was hampered by clients' struggles with adverse affective states such as boredom, anxiety, and depression (49).

Day-to-day risks of withdrawal, overdose, and economic precarity were and continue to be amplified by the pandemic, alongside exaggerated senses of repetitive time, a lack of temporal direction, and an inaccessibility of the future. Social distancing measures continue to deprive people who use drugs of the social relationships and sources of interaction that offer meaning and purpose. The intense isolation occasioned by the pandemic therefore exacerbates the boredom and frustration within which substance use often emerges, while heightening circumstances of social isolation that are associated with acute loneliness (25, 50, 51), senses of uselessness (29, 30), and hopelessness. In short, already fragile social and temporal structures have been further fragmented by the pandemic, as exemplified by Tamsin's testament to her fractious relationship with her friend, and her account of the present as both chaotic and unending.

Within this turbulent milieu, substances continue to offer a form of solace, enabling both breathing space and solitude. Substances provide a means of gaining distance from the present, in addition to enabling a heuristic of temporal relief that in turn allows for new affective-temporal possibilities. The intensified flux in substance availability and affordability, however, problematises even this aspect of everyday life, as further restrictions on choice produce unanticipated and harmful effects. Relatively recent trends in innovative and opportunistic ways of combining multiple substances have arguably laid the groundwork for adaptive responses to shifting drug markets.

Counteracting isolation among people who use drugs was recognized by the Scottish Government as a priority during the pandemic. The “Staying Connected Scotland Fund” provided tablet computers, smart phones, data SIM cards and subscriptions to teleconferencing services to enable participation in mutual aid and peer support groups (52). While this fund has been an invaluable way of bridging the “digital divide,” it is important to consider that a third of the population in Scotland lives alone, an even higher proportion among people who use drugs (53). This is compounded by many in this group being older with less engagement with online communications. Further, privacy when using online communications can be unattainable where people are street homeless or in shared temporary housing. Finally, we have no real idea of how long social distancing measures will need to be maintained, and we have evidence indicating that prolonged isolation is associated with 60–70% increase in mortality (54). In appreciation of this set of circumstances, clinicians and care-workers may now need to build in strategies to overcome isolation, balance the risks of isolation against the risk of exposure to Covid-19 and bridge the cultural unfamiliarity or discomfort with virtual social networking as a replacement for conventional human contact.

Our focus here on the disruption to interpersonal relationships among people who use drugs in Scotland speaks directly to the problems of internalized stigma and shame, which act as barriers to the formation of collective or community identities and ties. It is essential to recognize the impact of social capital, or the depth and extent of social networks, trust, and norms, as a protective factor against opioid overdose at the community level (55). To what extent will social isolation further increase community fragility, and through this increase the vulnerability of a group already beleaguered by the highest drug related death rate in the EU?

## Conclusion

Isolation and solitude, overall, comprise important – yet often overlooked or misconstrued – aspects of substance use, ones which take on particular significance in the context of the pandemic. Social relationships between people who use drugs are equally often mischaracterised as purely pragmatic or based predominantly on need, although there is increasing recognition that these, as with all relationships, are often grounded in bonds of care, love, trust, and solidarity (2, 4). Relationships need not be defined through substances, though substance use itself can produce forms of social, physical, and emotional intimacy that facilitate everyday survival (56). Circumstances of precarity, vulnerability and crises – such as the pandemic – can, however, serve to complicate and disrupt social relationships, exposing individuals to the harms of isolation. Intensified experiences of isolation during the pandemic have served to prompt heightened and often riskier substance use, which enables both a “shielding” against adverse affective states such as boredom, anxiety, despair, and trauma, and the pursuit of a desirable form of solitude. The isolation that has arisen from social distancing measures, and the collateral harms outlined above, have made such solitude all the more necessary and yet, with the disruption to daily life, all the more difficult to achieve.

Where isolation, boredom, and loneliness are problematic to the person who uses drugs, what then is the solution? As mentioned earlier, digital technology such as video-conferencing and virtual groups have been advanced as key interventions to mitigate the enforced isolation many are experiencing as a result of necessary social distancing. Virtual social support has doubtlessly been invaluable to some in reducing the impacts of isolation, though the ability to access virtual spaces is mediated by inequality and circumstance. There are similarly issues such as the loneliness paradox, where technology gives us the semblance of connectedness, without the substance of meaningful therapeutic relationships. Indeed, this has been observed by the authors in clinical practice where patient's expectations were raised through having access to a therapist virtually, only to be let down when they were unable to access the same therapist regularly. The current UK guidance on managing the isolation the pandemic has wrought is to stay connected and to access practical help, but with little indication of how this is to be done (57). For many people struggling with their substance use, following this advice would have been problematic pre-Covid-19. It is unlikely to be any easier now.

What remains clear is the necessity of attending to the complex dynamics of relatedness, isolation, and solitude that structure experiences of both substance use and social distancing. Fostering social connection and community surface as paramount in reducing the harmful aspects of isolation, though we argue that such approaches can be strengthened through a nuanced appreciation of the place that solitude occupies in substance user sociality. Addressing the impacts of the pandemic must begin with a full and dialogic engagement with lived experience and a commitment to involving those who use drugs in the design and implementation of policy and practice. In addition to the daunting and ever-present challenge of tackling systemic inequality and deprivation, the multitude of complex, diffuse, and often contradictory experiences must, somehow, be accounted for, both in responses to the pandemic and beyond.

## Data Availability Statement

The data analyzed in this study is subject to the following licenses/restrictions: Datasets comprised of ethnographic research are exempt from digital upload in University of St Andrews. Requests to access these datasets should be directed to Laura Roe, lr383@st-andrews.ac.uk.

## Ethics Statement

The studies involving human participants were reviewed and approved by University Teaching and Research Ethics Committee (UTREC) University of St Andrews. The patients/participants provided their written informed consent to participate in this study. Written informed consent was obtained from the individual(s) for the publication of any potentially identifiable images or data included in this article.

## Author Contributions

The ethnographic research that informs the paper was conducted by LR. LR, JP, JT, RI, SF, and AB contributed to the conception of the paper and made substantial contributions to the drafting and revision of the work. Each had final approval on the version for publication and agrees to be accountable for all aspects of the work.

## Conflict of Interest

The authors declare that the research was conducted in the absence of any commercial or financial relationships that could be construed as a potential conflict of interest.

## Footnotes

^1^Full ethical clearance was granted by the University of St Andrews' ethics committee for the research presented in this paper.

^2^All names and identifying features have been removed in order to protect the anonymity of the individuals who appear in this paper. All conversations and interviews presented were audio recorded with full written consent and permission was obtained for printing.

^3^‘Cannae' means ‘cannot' or ‘can't.'

^4^‘Ken' means ‘know' and is often used to mean ‘you know?'

^5^Tamsin went through periods of attempted abstinence, which she referred to as ‘keeping aff [off] it.'

^6^‘Vallies' is a slang term for Valium.

^7^During the lockdown, Roe contacted research participants by phone in order to keep in touch and maintain the research relationship while in-person fieldwork was impossible. The conversation with Tamsin in June was specifically audio recorded at her request, and excerpts are printed here with full consent.

^8^‘Fae' means ‘from' and ‘rattling' means ‘withdrawing.'
